# Spin-Orbit-Coupling-Governed Optical Absorption in Bilayer MoS_2_ via Strain, Twist, and Electric Field Engineering

**DOI:** 10.3390/nano15141100

**Published:** 2025-07-16

**Authors:** Lianmeng Yu, Yingliang Chen, Weibin Zhang, Peizhi Yang, Xiaobo Feng

**Affiliations:** 1 School of Physics and Electronic Information, Yunnan Normal University, Kunming 650500, China; 2133090006@ynnu.edu.cn (L.Y.); 220001@ynnu.edu.cn (W.Z.); 2 School of Mathematics and Information Technology, Lijiang Normal University, Lijiang 674100, China; 3 School of Energy and Environmental Science, Yunnan Normal University, Kunming 650500, China

**Keywords:** continuum model, twisted bilayer MoS_2_, strain, spin-orbit coupling, optical absorption

## Abstract

This paper investigates strain-, twist-, and electric-field-tuned optical absorption in bilayer MoS_2_, emphasizing spin-orbit coupling (SOC). A continuum model reveals competing mechanisms: geometric perturbations (strain/twist) and Stark effects govern one-/two-photon absorption, with critical thresholds (~9% strain, ~2.13° twist) switching spin-independent to spin-polarized regimes. Strain gradients and twist enhance nonlinear responses through symmetry-breaking effects while electric fields dynamically modulate absorption via band alignment tuning. By linking parameter configurations to absorption characteristics, this work provides a framework for designing tunable spin-resolved optoelectronic devices and advancing light–matter control in 2D materials.

## 1. Introduction

Since the discovery of graphene in 2004, its exceptional physical properties, including ultrahigh carrier mobility and optical transparency, have attracted extensive attention [1,2,3]. However, the zero-bandgap nature of graphene fundamentally limits its applications in optoelectronic devices. This limitation has driven the exploration of novel two-dimensional (2D) materials with tunable bandgaps, such as black phosphorus, transition metal dichalcogenides (TMDCs), and silicene [4,5]. Among these, TMDCs represented by MoS_2_ have emerged as promising candidates for next-generation electronics and photonics due to their semiconducting characteristics and strong spin-orbit coupling effects [6,7,8,9,10]. The sizeable bandgap of TMDCs effectively addresses graphene’s shortcomings in electronic and optoelectronic applications [11,12], positioning them as leading post-graphene materials for advanced device engineering [13,14,15,16,17].

The performance of optoelectronic devices critically depends on photon absorption efficiency and energy conversion capabilities, processes governed by interband transitions. This underscores the importance of precisely regulating the band structures of TMDCs to manipulate their optical absorption coefficients. The weak interlayer van der Waals interactions in TMDCs enable flexible structural modifications through twisting, straining, and electric field strength [18,19]. Unlike twisted graphene, where flat bands emerge only at specific “magic angles”, bilayer TMDCs exhibit flat band features across a broader angular range (<7°) [18,20], making them ideal platforms for achieving strong optical transition resonances [21,22]. Experimental studies have demonstrated that strain engineering can significantly enhance photoluminescence (PL) intensity in bilayer MoS_2_ [23] while localized strain gradients induce symmetry-breaking effects that generate pronounced nonlinear optical responses [24]. These findings suggest that the synergistic application of twist, strain, and electric fields could provide unprecedented control over MoS_2_’s optical absorption properties. Nevertheless, systematic theoretical investigations remain scarce regarding how these parameters, particularly under SOC considerations, influence one-photon absorption (OPA) and two-photon absorption (TPA) coefficients in MoS_2_ systems.

Addressing this critical knowledge gap, we employ a low-energy continuum model to comprehensively investigate the strain-, twist-, and electricfield strength-mediated modulation of OPA/TPA coefficients in bilayer MoS_2_ with the explicit consideration of SOC contributions. Our methodology involves the following: (1) calculating bandgap evolution under various strain ε, twist angle θ, and external electric field strength (E.F.) configurations; (2) applying second-order perturbation theory to quantify SOC-modified transition matrix elements; and (3) establishing quantitative relationships between external parameters and absorption coefficients through microscopic mechanism analysis. This work reveals three key advancements: first, the identification of competing modulation mechanisms between geometric perturbations (*θ*, *ε*) and Stark effects in controlling absorption characteristics; second, the discovery of critical thresholds (*ε*_c_ ≈ 9%, *θ*_c_ ≈ 2.13°) governing transitions between spin-independent and spin-polarized absorption regimes; third, the demonstration of quantum interference effects through Berry curvature engineering under strain gradients. These findings not only deepen our understanding of light–matter interactions in 2D materials but also establish a theoretical framework for designing tunable photonic devices with spin-resolved functionalities.

## 2. Theory

**Figure 1:**
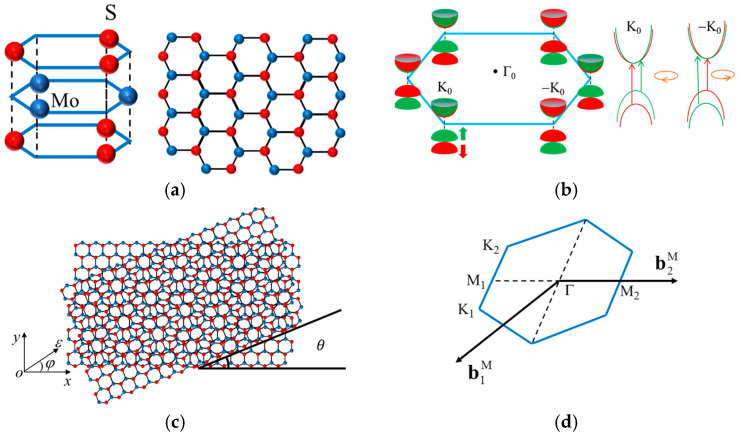
(**a**) Schematic of monolayer MoS_2_ crystal structure. Left: side view; right: top view. (**b**) Schematic drawing of the band structure at the band edges located at the K_0_ points. Green and red denote spin-up and spin-down states, respectively. (**c**) Formation of a moiré superlattice in twisted bilayer MoS_2_ and strain S with magnitude *ε* and direction *φ* application methodology. (**d**) Moiré Brillouin zone under compressive strain.

This paper focuses on MoS_2_ as a representative system of two-dimensional transition metal dichalcogenides MX_2_ (M = Mo/W; X = S/Se). Given their similar structural features and physical properties, the results could also be validated for other TMDCs. As illustrated in Figure 1a, monolayer MoS_2_ exhibits a hexagonal lattice structure, where each Mo atom is coordinated with six S atoms in a trigonal prismatic geometry, belonging to the D*_3h_* space group. The electronic structure, shown in Figure 1b, features Fermi levels located at two inequivalent valleys (K_0_ and −K_0_) in the Brillouin zone. While this valley degeneracy resembles graphene, MoS_2_ exhibits a distinct direct bandgap of approximately 1.66 eV. First-principles calculations combined with parameter fitting [25] reveal that the conduction band minimum (CBM) and valence band maximum (VBM) are primarily dominated by the Mo-derived *d*_3_*_z_*_^2^−_*_r_*_^2^_ and *d_x_*_^2^−_*_y_*_^2^_ ± *id_xy_* orbitals, respectively, with significant hybridization from the S atom *p*_x_ ± *ip*_y_ orbitals in both bands.

Compared to graphene, the electronic structure of MoS_2_ exhibits two notable characteristics. First, strong SOC arises from the metal d-orbital contributions. Specifically, the VBM at the K_0_ valley undergoes spin splitting with a magnitude of 2*λ* = 0.15 eV [25] while the CBM remains spin-degenerate due to the 2*dz*^2^ orbital symmetry (Figure 1b). Second, time-reversal symmetry enforces mirror-symmetric spin polarization between the K_0_ and −K_0_ valleys. To model these effects, we adopt a graphene-inspired staggered sublattice potential Hamiltonian [26,27] and incorporate SOC corrections originating from intra-atomic L·S interactions. The effective Hamiltonian for monolayer MoS_2_ is expressed thus:

(1) $$h(k)=at(\xi k_{x}\sigma_{x}+k_{y}\sigma_{y})+\frac{\Delta }{2}\sigma_{z}-\lambda \xi \frac{\left(\sigma_{z}-1\right)}{2}s_{z},$$

Here, *σ* denotes the Pauli matrices and valley index *ξ* = ±1. Key parameters include the lattice constant *a* = 3.193 Å, effective hopping integral *t* = 1.10 eV, bandgap Δ = 1.66 eV, and SOC strength *λ* = 0.075 eV (yielding 2*λ* = 0.15 eV splitting), which follows first-principles calculations [25]. Notably, the conservation of spin quantum number (*s*_z_ = ±1 for spin-up/down states) ensures the complete decoupling of spin channels in the Hamiltonian, significantly simplifying subsequent theoretical analyses.

We begin with a 2H-stacked bilayer MoS_2_ system and introduce strain and twist degrees of freedom to construct a deformed bilayer structure. As demonstrated in Figure 1c, when two 2D material layers exhibit a lattice-constant mismatch or twist angle, they form a moiré superlattice—a long-wavelength interference pattern with a periodicity much larger than the original atomic lattice spacing [18]. Let a_1_ = *a* (1, 0) and a_2_ = *a* (1/2, $\sqrt{3}$/2) denote the primitive lattice vectors, with corresponding reciprocal lattice vectors b_1_ = 2π/*a* (1, –1/$\sqrt{3}$) and b_2_ = 2π/*a* (0, 2/$\sqrt{3}$). The two inequivalent Dirac points in the first Brillouin zone are located at K_ξ_ = −*ξ*(4π/3*a*, 0). The combined deformation matrix U associated with the small angle rotation matrix *R*(*θ*) and the strain tensor *S*(*ε*, *φ*) is expressed thus [28,29]:

(2) $$\begin{array}{ll} U(\varepsilon ,\varphi ,\theta ) & =S(\varepsilon ,\varphi )+R(\theta )\\ & =\varepsilon \left(\begin{array}{cc} cos^{2}\varphi -\upsilon sin^{2}\varphi & (1+\upsilon )cos\varphi sin\varphi \\ (1+\upsilon )cos\varphi sin\varphi & sin^{2}\varphi -\upsilon cos^{2}\varphi \end{array}\right)+\left(\begin{array}{cc} 0 & -\theta \\ \theta & 0 \end{array}\right)\\ & =\left(\begin{array}{cc} \varepsilon_{xx} & \varepsilon_{xy}-\theta \\ \varepsilon_{xy}+\theta & \varepsilon_{yy} \end{array}\right) \end{array}$$

Here, υ = 0.25 [30] is the Poisson ratio. The introduction of strain and twist breaks the original lattice symmetry, modifying the lattice vectors $a_{i}^{l}$ and reciprocal vectors $b_{i}^{l}$ for *l* layer (*i* = 1, 2) as

(3) $$a_{i}^{l}=(I+U_{l})a_{i},b_{i}^{l}=(I-U_{l}^{T})b_{i},$$

where the relative deformation matrix satisfies *U*_2_ = −*U*_1_ = *U*/2 due to symmetry constraints. This deformation shifts the Dirac points and generates a moiré Brillouin zone, as illustrated in Figure 1d. The corresponding lattice vectors $a_{i}^{M}=a_{i}^{1}-a_{i}^{2}=U^{-1}a_{i}$ and reciprocal lattice vectors $b_{i}^{M}=b_{i}^{1}-b_{i}^{2}=U^{T}b_{i}$ of the moiré superlattice are derived accordingly. The strained Dirac point positions are determined by

(4) $$K_{\xi }=(I-U^{T})K_{0\xi }-\xi G,$$

where $G=\frac{\sqrt{3}}{2a}\beta (\varepsilon_{xx}-\varepsilon_{yy},-2\varepsilon_{xy})$; the effective gauge connection for the low energy Dirac fermions with the hopping modulus factor *β* ≈ 2.4 for MoS_2_ [31] represents the dimensionless hopping modulus factor characterizing the strain response of low-energy Dirac fermions.

The Hamiltonian of the bilayer MoS_2_ system adopts formalism analogous to that of bilayer graphene [28,29]:

(5)
H=
h
1
(
k)+
V
1(r
)
T
(
r
)
T†
(
r
)
h
2
(
k
)+V
2
(
r
),

Here, the intralayer coupling terms $h_{l}(k)=at[(I+U_{l}^{T}-\beta S_{l})(k-K_{l\xi })\cdot (\xi \sigma_{x},\sigma_{y})]+\frac{\Delta }{2}\sigma_{z}-\lambda \xi \frac{\left(\sigma_{z}-1\right)}{2}s_{z}$ are derived by applying the deformation matrix *U* to the monolayer Hamiltonian in Equation (1) [30]. The potential *V*_l_(**r**) accounts for moiré superlattice-induced intralayer modulations. Crucially, since the intralayer coupling parameters are orders of magnitude weaker than their interlayer counterparts, we set *V*_l_(**r**) = 0 in this model. The interlayer coupling term *T*(**r**) is expressed thus [28]:

(6) $$T(r)=\sum_{n=1}^{3}t(k)e^{ib_{n}^{M}\cdot r}$$

For computational implementation, we fix the interlayer spacing of bilayer MoS_2_ at 0.301 nm. The S-S interlayer hopping parameter *t*(**k**) is truncated at a maximum value of 10 meV to ensure computational tractability while preserving essential physical features [32].

When a uniform vertical electric field with strength E.F. is applied to the material, an interlayer energy offset Π = *ed*∙(E.F.) with electron charge *e* and *d* the thickness of the bilayer system is induced between the two layers. Consequently, the system Hamiltonian in Equation (5) requires modification by adding and subtracting Π/2 to and from the diagonal elements, respectively.

In crystalline solids, multiphoton absorption (MPA) processes can be described through time-dependent perturbation theory. For OPA, an electron in the ground state absorbs one photon of energy *ħ*ω and transitions to an excited state. The transition rate W_1_ for OPA in two-dimensional materials is derived from second-order perturbation theory as [33]

(7) $$W_{1}=\frac{2\pi }{\hbar }{\int \left|\langle \varphi_{f}\left|H_{int}\right|\varphi_{i}\rangle \right|}^{2}\delta (E_{f}-E_{i}-\hbar \omega )\frac{d^{2}k}{{\left(2\pi \right)}^{2}},$$

where *φ_i_* and *φ_f_* represent the initial and final state wavefunctions, *E_i_* and *E_f_* are their corresponding energies, and the Dirac delta function enforces energy conservation. The electron-radiation interaction Hamiltonian is *H*_int_ = e*ħ*/(*m_e_c*)A∙k, with effective mass *m_e_*, speed of light in vacuum *c*, and light wave vector potential A = A**e**. The OPA coefficient *α*_1_ relates to the transition rate *W*_1_ through

(8) $$\alpha_{1}=\frac{2W_{1}\hbar \omega }{Id},$$

where *I* = *ε*_ω_^1/2^ω^2^A^2^(2π*c*)^−1^ is the incident light intensity; *ε*_ω_ the optical-frequency dielectric constant. For TPA, the process involves sequential absorption of two photons via intermediate states. The TPA transition rate *W*_2_ is expressed as [33]

(9) $$W_{2}=\frac{2\pi }{\hbar }{\int \left|M_{f,i}\right|}^{2}\delta (E_{f}-E_{i}-2\hbar \omega )\frac{d^{2}k}{{\left(2\pi \right)}^{2}},\;M_{f,i}=\sum_{m}\frac{\langle \varphi_{f}\left|H_{int}\right|\varphi_{m}\rangle \langle \varphi_{m}\left|H_{int}\right|\varphi_{i}\rangle }{E_{m}-E_{i}-\hbar \omega },$$

where *φ_m_* and *E_m_* correspond to intermediate states and energies. The TPA coefficient *α*_2_ follows:

(10) $$\alpha_{2}=\frac{2W_{2}\hbar \omega }{I^{2}d}$$

## 3. Results and Discussion

### 3.1. Band Structure Modulation Mechanisms

In photon absorption processes, the probability and efficiency of electron transitions from the valence band to the conduction band are fundamentally governed by the material’s band topology. Therefore, a systematic investigation of the synergistic modulation effects induced by strain magnitude *ε*, twist angle *θ*, and external electric field strength E.F. on the band structure is critical. Guided by the *D*_3_*_h_* point group symmetry of the system, we focus on the electronic state evolution at the *ξ* = −1 valley.

**Figure 2:**
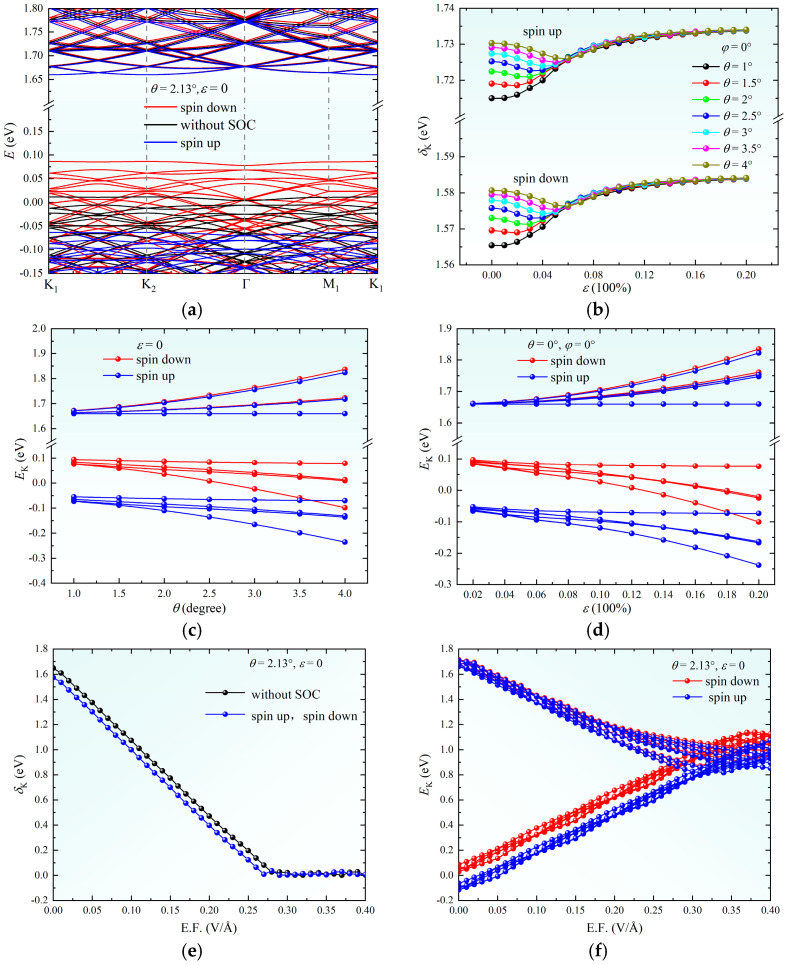
(**a**) Band structure of twisted bilayer MoS_2_. (**b**) Strain dependence of bandgap at the **K** point for different twist angles *θ*. (**c**) Energy of bands near the Fermi level at the **K** point versus *θ* under zero strain. (**d**) Energy of bands near the Fermi level at the **K** point versus *ε* for untwisted bilayer MoS_2_ under uniaxial strain *φ* = 0°. (**e**) Bandgap at the **K** point near the Fermi level versus electric field strength (E.F.) for *θ* = 2.13° without strain. (**f**) Energy of bands near the Fermi level at the K point versus *ε* for *θ* = 2.13° without electric field.

As illustrated in Figure 2a, SOC induces spin-polarized splitting at the VBM of the twisted bilayer system, forming spin-up and spin-down subbands with an energy splitting of Δ_SOC_ ≈ 0.15 eV. This phenomenon arises from the SOC-mediated orbital-momentum locking effect, which lifts the spin degeneracy by breaking spatial inversion symmetry [20]. The bandgap *δ*_k_ at the **K** point exhibits distinct nonlinear behavior under the combined modulation of strain magnitude *ε* and twist angle *θ* (Figure 2b). In the low-strain regime (*ε* < 6%), *δ*_k_ increases monotonically with *θ*, attributed to enhanced wavefunction localization caused by the attenuation of interlayer orbital coupling strength. In the high-strain regime (*ε* ≥ 6%): *δ*_k_ becomes robust against *θ* variations due to strain-driven lattice relaxation dominating band renormalization processes. The energy bands near the Fermi level undergo significant restructuring under *θ* and *ε* modulation (Figure 2c,d). This originates from the spatial confinement effects of moiré superlattice potentials: Increasing *θ* enhances wavefunction localization within moiré periodic potentials while *ε* modifies Brillouin zone symmetry to shift van Hove singularity positions. Notably, these geometric perturbations primarily redistribute carriers rather than altering the intrinsic bandgap, enabling the independent control of optical absorption spectra and electronic band alignment.

In contrast, vertical electric fields modulate *δ*_k_ through Stark-effect-driven interlayer charge transfer (Figure 2e). *δ*_k_ decreases linearly with external electric field strength, culminating in a semiconductor-to-metal transition at 0.29 V/Å, where interlayer tunneling dominates transport [34,35,36]. When field increases to 0.32 V/Å, this confirms SOC-enhanced field sensitivity via effective mass reduction [20]. This dimension-dependent regulatory disparity highlights distinct mechanisms: electric fields directly modify band alignment through electrostatic potentials while geometric perturbations (*θ*, *ε*) govern electronic correlations via orbital hybridization tuning.

### 3.2. Single-Photon Absorption Coefficient

**Figure 3:**
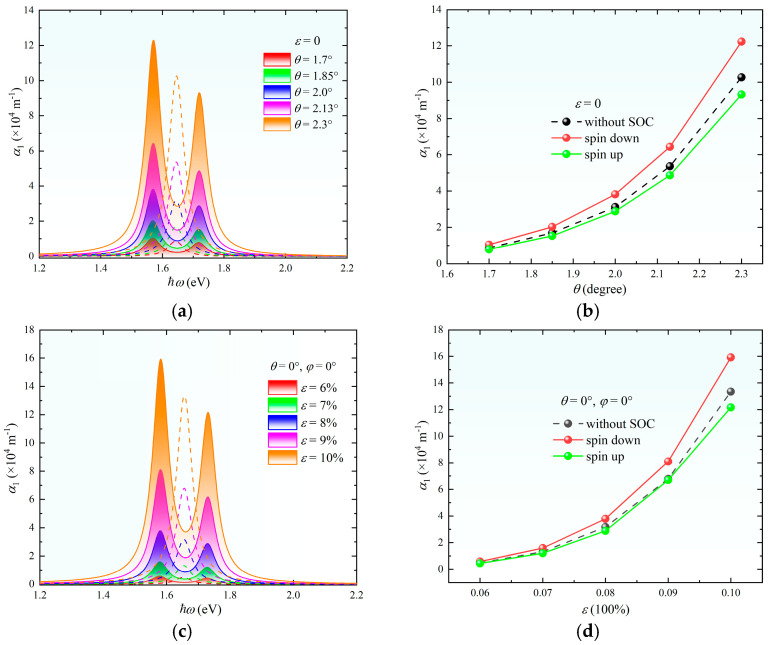
(**a**) One-photon absorption spectra of MoS_2_ at different twist angles *θ* under zero strain. (**b**) Peak values of the one-photon absorption coefficient versus *θ* without strain. (**c**) One-photon absorption spectra under isotropic strain *φ* = 0° at varying strain magnitudes *ε* without twisting. (**d**) Peak values of the one-photon absorption coefficient versus *ε* without twisting. *Note:* In (**a**–**c**), the left and right peaks correspond to spin-down and spin-up absorption, respectively, while the dashed central peak represents the spin-independent case (without SOC).

As shown in Figure 3, the SOC induces the characteristic splitting of the one-photon absorption coefficient α_1_ in twisted bilayer MoS_2_, generating dual absorption peaks flanking the SOC-free central peak. This phenomenon originates from the spin-selective transitions governed by the hybridization of Mo’s *d_x_*_^2^ −_ *_y_*_^2^_ ± *idxy* orbitals at the valence band maximum, which lifts spin degeneracy through broken inversion symmetry [37]. The absorption coefficient reaches magnitudes of 10^5^ m^−1^, with both peak intensity and spectral width exhibiting strong dependence on twist angle *θ* and strain *ε*. Specifically, increasing θ from 1.7° to 2.3° enhances α_1_ by amplifying the moiré-potential localization effect-reduced interlayer coupling strengthens wavefunction confinement, thereby increasing the density of states near van Hove singularities (Figure 2c,d). This geometric modulation simultaneously widens absorption peaks (FWHM expansion > 30%) due to enhanced band nesting effects. Strain engineering (*ε* > 6%) further elevates α_1_ through Brillouin-zone-compression-induced band restructuring [28], which modifies momentum conservation rules and activates normally forbidden interband transitions. Notably, spin-down absorption channels dominate under high *θ*/*ε* conditions, approaching the absorption intensity of SOC-free systems (Figure 3d), a consequence of strain-mediated C_3_ symmetry breaking that preferentially enhances dipole matrix elements for spin-down transitions. The decoupled control capabilities, *θ* controlling spin polarization via spatial carrier localization and *ε* tuning spectral response through symmetry relaxation, establish a dual-parameter strategy for designing polarization-sensitive photonic devices with customized absorption profiles.

**Figure 4:**
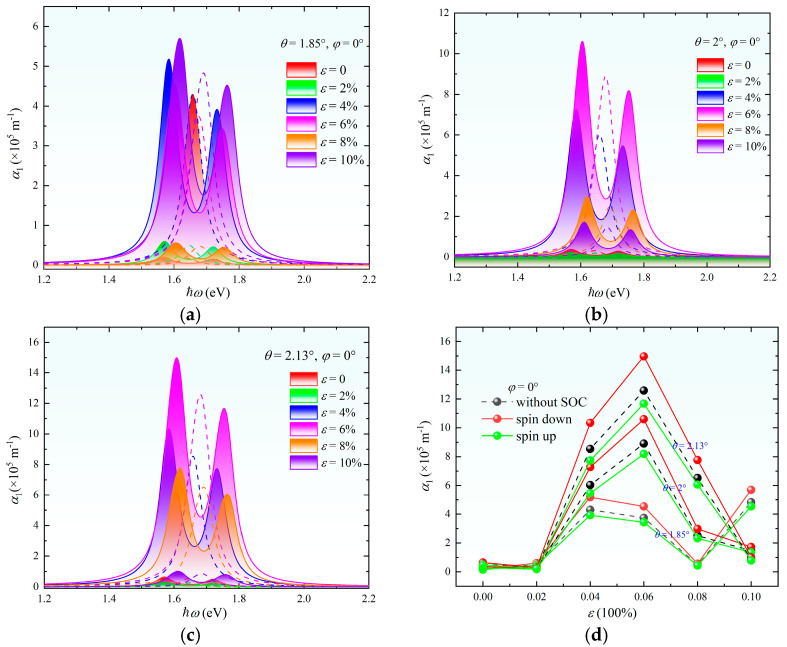
(**a**–**c**) One-photon absorption spectra of twisted bilayer MoS_2_ under varying strain magnitudes *ε* at twist angles of *θ* = 1.85° (**a**), *θ* = 2.00° (**b**), and *θ* = 2.13° (**c**). The left and right peaks correspond to spin-down and spin-up absorption channels while the dashed central peak represents the case without SOC. (**d**) Peak values of the one-photon absorption coefficient versus *ε* at the three twist angles.

Systematic investigations reveal a nonlinear evolution of the one-photon absorption coefficient α_1_ in bilayer MoS_2_ across the strain-twist angle parameter space (Figure 4). For systems with twist angles of 1.85°, 2°, and 2.13°, α_1_ exhibits a biphasic response as *ε* increases from 0% to 10%, initial enhancement followed by suppression. The critical strain *ε*_c_ corresponding to peak α_1_ demonstrates *θ*-dependent shifts: *ε*_c_ = 10% for *θ* = 1.85° while *ε*_c_ = 6% for *θ* = 2° and 2.13° (Figure 4d). This phenomenon originates from the competing modulation effects of strain and the twist angle on interlayer coupling—strain enhances transition matrix elements through Brillouin zone compression whereas excessive strain (*ε* > *ε*_c_) disrupts interlayer orbital hybridization, leading to suppressed absorption. The observed angular dependence of *ε*_c_ highlights the geometric frustration between moiré periodicity and lattice deformation at different twist configurations.

**Figure 5:**
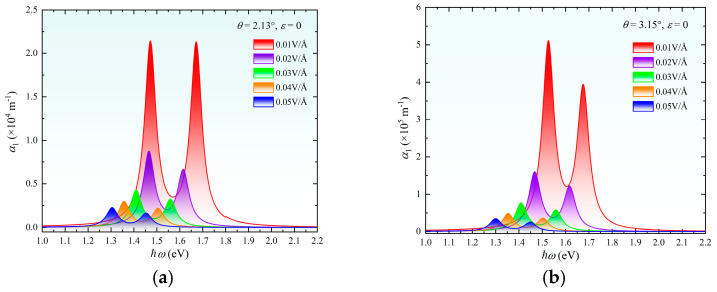
One-photon absorption spectra of twisted bilayer MoS_2_ under electric fields ranging from 0 to 0.05 V/Å. (**a**) *θ* = 2.13°; (**b**) *θ* = 3.15°.

The one-photon absorption coefficient exhibits exceptional sensitivity to vertical electric fields, as demonstrated in Figure 5. Upon the application of E.F. = 0.1 V/Å, α_1_ decreases by two orders of magnitude (from 10^6^ cm^−1^ to 10^4^ cm^−1^) accompanied by a pronounced redshift. This phenomenon originates from the interlayer Stark effect induced by the electric field, where the theoretical prediction of redshift magnitude aligns closely with experimental observations [20]. The bandgap reduction (Figure 2f) lowers the photon energy required for resonant transitions, resulting in systematic absorption peak redshift. Concurrently, bandgap narrowing significantly suppresses the interband transition probability. Here, electric-field-induced wavefunction delocalization attenuates the momentum matrix element while the density of states within individual bands (conduction/valence) remains stable (Figure 2d). These combined effects drive the exponential attenuation of α_1_ with an increasing external electric field.

### 3.3. Two-Photon Absorption Coefficient

**Figure 6:**
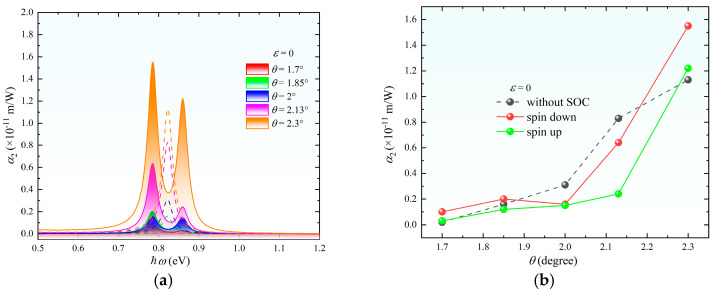

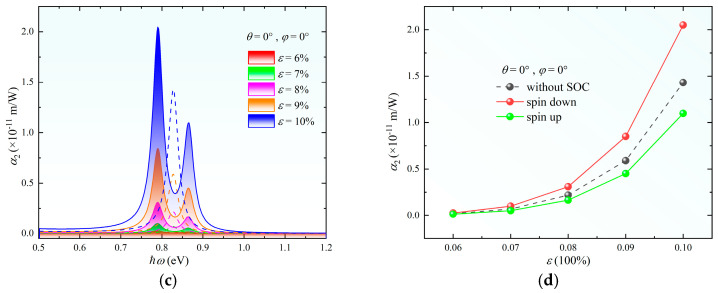
(**a**) Two-photon absorption spectra of MoS_2_ at different twist angles *θ* without strain. (**b**) Peak values of the two-photon absorption coefficient versus *θ* without strain. (**c**) Two-photon absorption spectra under isotropic strain *φ* = 0° at varying strain magnitudes *ε* without twisting. (**d**) Peak values of the two-photon absorption coefficient versus *ε* without twisting. *Note:* In (**a**–**c**), the left and right peaks correspond to spin-down and spin-up absorption, respectively, while the dashed central peak represents the case without SOC.

The two-photon absorption coefficient α_2_ exhibits unique response characteristics under the modulation of strain or twist angle, as shown in Figure 6. Distinct from one-photon absorption, α_2_ undergoes a critical transition at *θ* = 2°, where spin-down and spin-up absorption intensities become equal while both remain lower than the spin-independent case. Beyond this critical angle, pronounced spin polarization emerges with an increasing *θ*. This phenomenon originates from the competition between interlayer coupling strength and moiré superlattice potentials. Strain modulation demonstrates a threshold-dependent behavior. For *ε* < 6%, spin-up, spin-down, and spin-independent absorption coefficients remain comparable. When *ε* > 6%, α_2_ increases linearly with strain, accompanied by enhanced spin polarization. The observed absorption peak broadening correlates directly with the density of states dispersion shown in Figure 2, confirming that geometric perturbations amplify interband scattering rates, thereby expanding the two-photon resonance window. This synergistic control of spectral broadening and spin polarization establishes new degrees of freedom for developing ultrafast spintronic–photonic devices.

**Figure 7:**
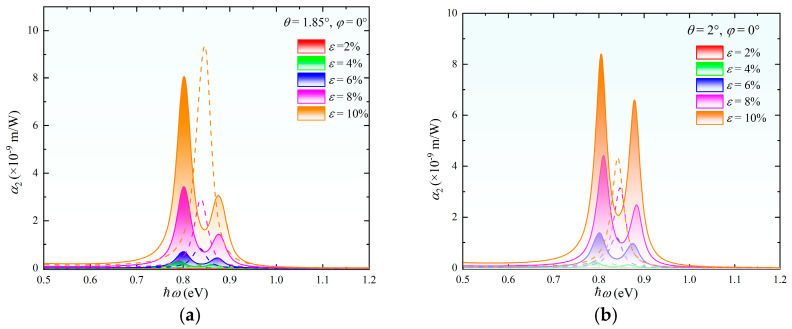

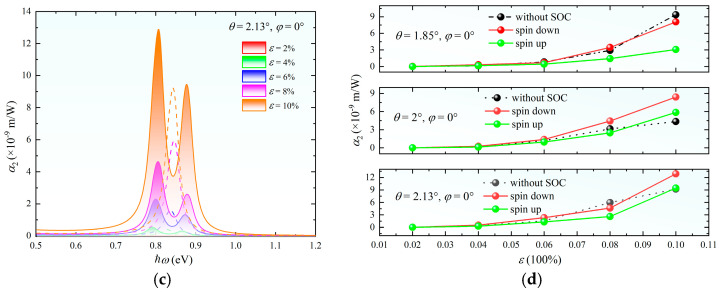
Two-photon absorption spectra of twisted bilayer MoS_2_ under varying strain magnitudes ε at twist angles of *θ* = 1.85° (**a**), *θ* = 2.00° (**b**), and *θ* = 2.13° (**c**). The left and right peaks correspond to spin-down and spin-up absorption channels while the dashed central peak represents the case without SOC. (**d**) Peak values of the two-photon absorption coefficient versus *ε* at the three twist angles.

The two-photon absorption coefficient α_2_ exhibits multi-extremal response characteristics under the synergistic modulation of strain and the twist angle, as illustrated in Figure 7. For the system with *θ* = 1.85° and *ε* = 10%, the spin-independent α_2_ reaches a maximum value of 9 × 10^−9^ m/W, attributed to strain-enhanced interlayer hybridization. Notably, at *θ* = 2°, the spin-down absorption channel matches the spin-independent case when *ε* = 8%, whereas increasing *ε* to 10% causes the spin-up absorption to surpass the spin-independent value. This reveals the strain-mediated preferential enhancement of specific spin channels through C_3_ symmetry breaking. For the *θ* = 2.13° system, spin-independent absorption dominates at *ε* = 8%, but at *ε* = 10%, the spin-up absorption equals the spin-independent value while remaining lower than the spin-down counterpart. This indicates a critical strain threshold of approximately 9%, beyond which spin-polarized absorption prevails. Full parameter-space analysis demonstrates the systematic enhancement of α_2_ across the 1375–1649 nm wavelength range with increasing *ε*. This strain–twist synergy establishes a theoretical framework for designing wavelength-tunable two-photon detectors. Optimal absorption at the 1550 nm telecommunication window can be achieved through strategic *θ-ε* parameter matching.

**Figure 8:**
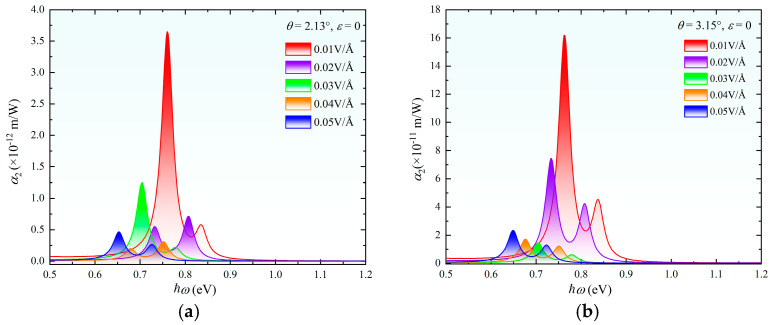
Two-photon absorption spectra of twisted bilayer MoS_2_ under zero strain with electric fields ranging from 0 to 0.05 V/Å: (**a**) *θ* = 2.13°; (**b**) *θ* = 3.15°.

Figure 8a,b illustrate the evolution of the two-photon absorption coefficient α_2_ with vertical electric field strength in twisted bilayer MoS_2_ systems at angles of *θ* = 2.13° and 3.15°. The pronounced modulation of the bandgap by electric field drives a highly nonlinear response in α_2_. As external electric field increases from 0.01 to 0.05 V/Å, α_2_ decreases by approximately one order of magnitude (e.g., from 3.6 × 10^−12^ m/W to 0.5 × 10^−12^ m/W for *θ* = 2.13°), accompanied by a redshift of the absorption peak. Notably, the attenuation of α_2_ exhibits nonmonotonic behavior—an anomalous enhancement observed at specific electric field strength values. This phenomenon is attributed to the electric-field-driven redistribution of electron density between layers, which modifies the energy distribution of intermediate states in the two-photon transition matrix element. Furthermore, the redshift rate correlates strongly with the bandgap contraction rate, consistent with theoretical predictions of the Stark effect [20]. We have compiled and compared the currently reported experimental and theoretical data on two-photon absorption in MoS_2_ (see Table 1), providing a reference for related experimental studies and practical applications.

## 4. Conclusions

This paper has comprehensively investigated the synergistic modulation of optical absorption properties in bilayer MoS_2_ under strain, the twist angle, and vertical electric fields, with the explicit consideration of spin-orbit coupling (SOC) effects. The interplay between geometric perturbations (strain and twist) and Stark effects reveals competing mechanisms for controlling spectral response and spin polarization. Critical thresholds (*ε*_c_ ≈ 9%, *θ*_c_ ≈ 2.13°) govern transitions between spin-independent and spin-polarized absorption regimes, driven by strain-induced Brillouin zone compression and twist-mediated moiré potential localization. Strain gradients further enhance nonlinear optical responses through symmetry-breaking effects while vertical electric fields enable the dynamic tuning of absorption coefficients over orders of magnitude by renormalizing bandgaps and delocalizing wavefunctions. These findings establish bilayer MoS_2_ as a versatile platform for spin-resolved optoelectronics, offering tailored absorption profiles through parameter-specific strain–twist–electric field combinations. The theoretical framework presented here advances the design of tunable photonic devices such as polarization-sensitive detectors and wavelength-selective nonlinear optical modulators. Future efforts should prioritize the experimental validation of predicted thresholds and explore strain-gradient-engineered quantum interference in ultrafast spintronic applications, bridging theoretical insights with practical device engineering.

## Figures and Tables

**Table 1:** TPA coefficient of MoS_2_ using this theory and other theories, as well as experimental data for comparison.

Thickness	Electric Field (V/Å)	Twist Angle (Degree)	Strain Magnitude (%)	Wavelength (nm)	β (Experiment) (m/W)	β (Other Theory) (m/W)	β (This Theory) (m/W)
ML-MoS_2_	0	0	0	1030	(7.62 ± 0.15) × 10^−8^ [38]		
ML-MoS_2_	0	0	0	800		8 × 10^−9^ [39]	
ML-MoS_2_	0	0	0	780		7.47 × 10^−11^ [40]	
ML-MoS_2_	0	0	0	1030		4.2 × 10^−11^ [40]	
50.0 ± 0.75 (nm)	0	0	0	1030	(4.99 ± 0.02) × 10^−9^ [41]		
25–27 layers	0	0	0	800	(6.6 ± 0.4) × 10^−10^ [42]		
25–27 layers	0	0	0	1030	(1.14 ± 4.3) × 10^−10^ [42]		
BL-MoS_2_	0	1.7	0	1506 ± 60			0.1 × 10^−11^
BL-MoS_2_	0	2.3	0	1506 ± 60			1.55 × 10^−11^
BL-MoS_2_	0	0	6	1506 ± 60			0.1 × 10^−11^
BL-MoS_2_	0	0	10	1506 ± 60			2.0 × 10^−11^
BL-MoS_2_	0	1.85	2	1469 ± 60			0.1 × 10^−9^
BL-MoS_2_	0	1.85	10	1469 ± 60			0.85 × 10^−9^
BL-MoS_2_	0.01	2.13	0	1627			3.62 × 10^−12^
BL-MoS_2_	0.05	2.13	0	1902			0.46 × 10^−12^
BL-MoS_2_	0.01	3.15	0	1623			1.62 × 10^−11^
BL-MoS_2_	0.05	3.15	0	1914			2.27 × 10^−11^

## Data Availability

The original contributions presented in this study have been included in the article. Further inquiries can be directed to the corresponding author.

## Abbreviations

The following abbreviations have been used in this manuscript:

2D	two-dimensional
TMDCs	transition metal dichalcogenides
SOC	spin-orbit coupling
PL	photoluminescence
OPA	one-photon absorption
TPA	two-photon absorption
MPA	multi-photon absorption
CBM	conduction band minimum
VBM	valence band maximum
ML-MoS_2_	monolayer MoS_2_
BL-MoS_2_	bilayer MoS_2_
